# Toward 70% cervical cancer screening coverage: Technical challenges and opportunities to increase access to human papillomavirus (HPV) testing

**DOI:** 10.1371/journal.pgph.0001982

**Published:** 2023-08-16

**Authors:** Kathryn A. Kundrod, Jose Jeronimo, Beatrice Vetter, Mauricio Maza, Gad Murenzi, Natacha Phoolcharoen, Philip E. Castle

**Affiliations:** 1 Division of Cancer Epidemiology and Genetics, National Cancer Institute, Rockville, Maryland, United States of America; 2 FIND, the Global Alliance for Diagnostics, Geneva, Switzerland; 3 Department of Noncommunicable Diseases and Mental Health, Unit of Noncommunicable Diseases, Violence and Injury Prevention, Pan American Health Organization, Washington, DC, United States of America; 4 Einstein-Rwanda Research and Capacity Building Program, Research for Development Rwanda and Rwanda Military Hospital, Kigali, Rwanda; 5 Faculty of Medicine, Department of Obstetrics and Gynecology, Chulalongkorn University, King Chulalongkorn Memorial Hospital, Bangkok, Thailand; 6 Division of Cancer Prevention, National Cancer Institute, Rockville, Maryland, United States of America; The University of Texas Health Science Center at Houston School of Public Health - San Antonio Campus, UNITED STATES

## Abstract

The World Health Organization (WHO) has called for the elimination of cervical cancer as a public health problem. Cervical cancer screening through human papillomavirus (HPV) testing is a core component of the strategy for elimination, with a set target of screening 70% of women twice in their lifetimes. In this review, we discuss technical barriers and opportunities to increase HPV screening globally.

## Introduction

Cervical cancer remains a major cause of female morbidity and mortality globally, with an estimated 604,000 new cases and 342,000 deaths in 2020 [1]. Almost 90% of cervical cancers and related deaths occur in low- and middle-income countries (LMICs) [1]. Sexual transmission of 12–15 carcinogenic human papillomavirus (HPV) genotypes (types) cause virtually all cervical cancer globally [2].

Cervical cancer is preventable through prophylactic HPV vaccination of young women, most effectively in those who have not yet acquired HPV, and screening with treatment of screen positives with pre-cancers in mid-adult women. In 2018, the World Health Organization (WHO) announced a global call for action to eliminate cervical cancer with the target of having 90% of girls fully vaccinated by age 15, 70% of women screened, and 90% of women with pre-cancer or invasive cancer managed. Currently, many LMICs are not poised to achieve the WHO screening target. For example, no country in sub-Saharan Africa (SSA) has achieved the screening target [3], the average screening coverage in SSA is 4% [4], and most screenings are conducted using a low-performance test. Most countries in Latin America and Asia have less than 50% screening coverage [3], with regional screening coverages at 29% in Latin America and the Caribbean, 11% in Western Asia, 4% in Central and Southern Asia, and 13% in Eastern and South-Eastern Asia [4]. Some of the same strategies to implement cervical cancer prevention and control programs, importantly, could be useful in areas with high health disparities in high-income countries, as well.

Testing for carcinogenic HPV (“HPV testing”), the etiologic agent that causes virtually all cervical cancer, is the more sensitive, reliable screening method compared to cytology (i.e., Pap test) or visual inspection with acetic acid (VIA) (Table 1) [2]. HPV testing more effectively identifies individuals who are at risk of or have already developed cervical pre-cancer or cancer [5,6]. HPV DNA testing is the WHO-recommended screening test although qualitative HPV mRNA testing is suggested as an alternative where there is capacity and only for women who are not living with HIV [6].

**Table 1:** Comparison of cervical cancer screening methods [2].

	Carcinogenic HPV testing	Cytology (Pap)	Visual Inspection with Acetic Acid (VIA)
Brief definition	Molecular testing for carcinogenic HPV, which is the etiologic cause of cervical cancer	Microscopic evaluation of cervical cells to look for abnormalities associated with cervical cancer	Visual inspection of the cervix to look for lesions, which turn white in the presence of 5% acetic acid
General testing logistics	Sample is self-collected or provider-collected during a pelvic exam and sent to a laboratory for testing	Cells are collected from the cervix by a provider during a pelvic exam and sent to a laboratory for evaluation	Provider applies dilute acetic acid during a pelvic exam and evaluates the cervix with adequate illumination
Infrastructure required	Medium	High	Low
Sensitivity	High	Medium	Variable
Specificity	Medium	High	Variable

Although the evidence indicates that HPV detection is the most effective method of cervical cancer screening, HPV testing remains largely inaccessible in LMICs, where most of the global burden of cervical cancer resides. Of the 47 countries in SSA, national screening policies exist for 15 countries; but only two countries, Botswana, and Rwanda, have national screening policies that include HPV DNA testing [3]. However, the ability to screen with HPV DNA tests is still contingent upon test—and testing—availability and budget for procurement.

**Fig 1:**
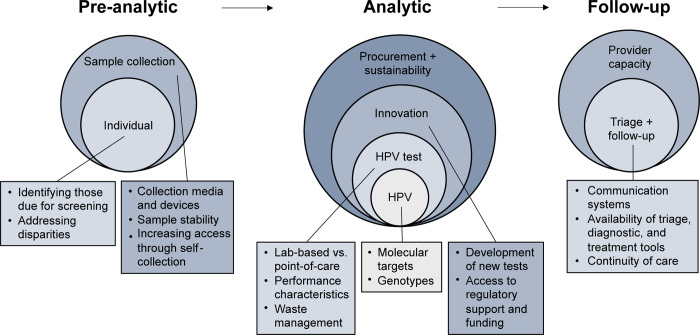
Framework for review of HPV technical barriers.

Thus, to overcome this global health disparity in cervical cancer, measures need to be taken to increase the access and availability of validated HPV tests and testing protocols for cervical cancer screening. Here, we provide background on pre-analytic and analytical aspects of HPV testing, and we then consider technical barriers and opportunities related to universal access HPV testing for cervical cancer screening aligned with the framework shown in Fig 1.

### Background on pre-analytic and analytic aspects of HPV testing

HPV testing requires sample collection, stable sample storage and transport, and molecular testing by trained laboratorians. Technical considerations related to pre-analytic and analytic phases of testing are discussed below.

#### 1.1 Screening

HPV testing may use a provider-collected specimen with or without pelvic exam or a self-collected cervicovaginal specimen. With self-collection, the sample is collected with a swab and is stored in a tube either with liquid sample collection media or without (i.e., dry) until analysis. Samples must be transported to a lab for HPV testing and subsequent triage testing of HPV-positive specimens, which requires transport infrastructure, trained laboratory personnel, equipment, and reagents.

#### 1.2 HPV test performance evaluation

As of January 2020, there were 254 distinct HPV tests on the global market and 425 HPV test variants (i.e., targets different HPV genotypes with the same essential testing technology) [7]. Even though 41% of them had peer-reviewed publications, 81.8% had no publications demonstrating analytical or clinical evaluation in a peer-reviewed journal, over 90% did not have performance evaluations in accordance with standards agreed upon in the HPV community, and only five had US Food and Drug Administration (FDA) approval for HPV testing alone or for co-testing with cytology [7]. Tests on the market detect a variety of molecular targets, report results with varying levels of genotyping information, and have a wide range of throughputs with accompanying differences in infrastructure needs (Table 2). In this section, HPV test considerations are discussed.

**Table 2:** Summary of HPV testing platforms.

Test mfgr.	Platform	Assay	CE IVD, WHO PQ, US FDA [56,57]	Target	HPV genotypes reported		8-hour through-put	Repro-ducibility[58] *	Level of validation [58] *
					*Individual*	*Pooled*			
Abbott	m2000 sp/rt	*Real*Time High Risk (HR) HPV	CE, WHO	L1 DNA	16, 18	31, 33, 35, 39, 45, 51, 52, 56, 58, 59, 66, 68	96	++	+++
Abbott	Alinity m	Alinity m High Risk (HR) HPV	CE	L1 DNA	16, 18, 45	pool 1: 31, 33, 52, 58, pool 2: 35, 39, 51, 56, 59, 66, 68	NR	++	++
Hologic	Panther	Aptima HPV	CE, FDA	E6/E7 mRNA	*standard assay*: none	*standard assay*: 16, 18, 31, 33, 35, 39, 45, 51, 52, 56, 58, 59, 66, 68	275	++	NR against DNA comparator
					*genotyping assay*: 16**	*genotyping assay*: 18/45**			
Hologic	thermal cycler + plate reader	Cervista HPV 16/18 assay	FDA	L1, E6, E7 DNA	16, 18	none	NR	NR	+
Qiagen	careHPV Test System	careHPV	CE, WHO	DNA	none	16, 18, 31, 33, 35, 39, 45, 51, 52, 56, 58, 59, 66, 68	Up to 270	NR	-
Qiagen	Modular system	Digene Hybrid Capture 2	CE, FDA	Whole genome	none	16, 18, 31, 33, 35, 39, 45, 51, 52, 56, 58, 59, 68	Up to 352	NR	comparator
Roche	4800/6800/ 8800	cobas HPV	CE, FDA	L1 DNA	16, 18	31, 33, 35, 39, 45, 51, 52, 56, 58, 59, 66, 68	192/384/ 960	++	+++
BD	Viper LT System	Onclarity HPV Assay	CE, FDA	E6/E7 DNA	16, 18, 31, 45, 51, 52	pool 1: 33, 58, pool 2: 56, 59, 66, pool 3: 35, 39, 68	90	++	+++
Cepheid	GeneXpert (IV/ XVI/Infinity-48/Infinity-80)	Xpert HPV	CE, WHO	E6/E7 DNA **	16**	pool 1: 18/45, pool 2: 31, 33, 35, 52, 58 pool 3: 51, 59 pool 4: 39, 56, 66, 68a **	32/128/ 384/640	++	++
Life-River Biotech	Aurtrax extractor + Life 96 PCR System	Harmonia HPV, Venus HPV kit	CE	DNA	16, 18 (Harmonia only)	31, 33, 35, 39, 45, 51, 52, 56, 58, 59, 66, 68 (16, 18 included for Venus)	384	NR	NR
Quan-Dx	thermal cycler	MeltPro High Risk HPV Genotyping Assay	CE	DNA	16, 18, 31, 33, 35, 39, 45, 51, 52, 56, 58, 59, 66, 68	none	NR	NR	NR
Atila Bio- systems	thermal cycler	Ampfire HPV	CE**	DNA (multiple regions) **	*genotyping assay*: 16, 18, 31, 33, 35, 39, 45, 51, 52, 53, 56, 58, 59, 66, 68	*genotyping assay*: none	NR	NR	NR
					*partial genotyping assay*: 16, 18 **	*partial genotyping assay*: 31, 33, 35, 39, 45, 51, 52, 53, 56, 58, 59, 66, 68 **			
Gen-omica	NEDxA	CLART HPV 4s **	CE**	E6/E7 DNA **	16, 18, 31, 33, 35, 39, 45, 51, 52, 56, 58, 59, 66, 68 (+ low-risk types 6, 11) **	none **	NR	++	+
Molbio	Truelab PCR analyzer	Truenat HPV-HR	CE**	E6/E7 DNA **	none **	16, 18, 31, 45 **	NR	NR	NR
See-Gene **	NIMBUS/ STARlet, thermal cycler**	Anyplex II HPV HR Detection **	CE**	L1 DNA **	16, 18, 31, 33, 35, 39, 45, 51, 52, 56, 58, 59, 66, 68**	none **	184 **	++	+++

Source: UNITAID Cervical Cancer Technology Landscape [8], unless otherwise indicated

* Reproducibility evaluation: +: High intra-laboratory reproducibility, ++: High inter- and intra-laboratory reproducibility; Validation evaluation as proposed by Meijer et al, 2009 [59], and employed by Arbyn et al, 2021 [58]: -: Test did not reach validation criteria, +: Partially validated, ++: Fully validated in one study, +++: Fully validated in multiple studies.

** Updated from UNITAID landscape based on manufacturer report (S1 Table). *NR*: *Not reported*.

*1*.*2*.*1 HPV molecular targets*. Commercial, validated HPV tests detect the DNA or RNA of HPV16, 18, 31, 33, 35, 39, 45, 51, 52, 56, 58, 59, and/or 68, which are considered carcinogenic or probably carcinogenic [2]. Most commercial HPV tests detect those 13 carcinogenic genotypes, though some detect fewer, and some unfortunately target HPV53 and/or 66 that rarely cause cancer but greatly increase test positivity and decrease clinical specificity [8,9].

Most HPV tests on the market detect HPV DNA. Persistence of an HPV infection and progression toward high-grade disease will lead to the overexpression of HPV early viral proteins, e.g., oncoproteins E6 and E7 [10]. Therefore, quantitative HPV mRNA testing could theoretically confer greater specificity of screening, providing information on both the presence of an HPV infection and progression toward high-grade disease. Currently, there is one HPV mRNA test in widespread use, Aptima HPV (Hologic), which provides qualitative results on the presence of or absence of HPV mRNA. Recently, WHO indicated Aptima HPV is an acceptable substitute for HPV DNA testing among women who are not living with HIV and with provider-collected cervical samples only [6].

*1*.*2*.*2 HPV Genotyping*. Pooled HPV DNA testing reports a positive or negative readout for the presence of any carcinogenic HPV genotype, which is appropriate for screening per WHO guidelines [2]. However, there is significant variability in the carcinogenicity of each type. HPV16 and 18 are the most carcinogenic and account for over 70% of cervical cancer cases, and HPV31, 33, 45, 52, and 58 cause another 20% of cervical cancers [11]. At the other end, HPV68 is only considered “probably” carcinogenic, and several HPV types, including HPV53 and 66, are considered “possibly” carcinogenic [12].

Most HPV tests on the market identify some HPV genotypes individually. Recent evidence indicates genotyping is clinically useful for risk determination, either by categorizing genotypes into risk strata or through tracking single-genotype persistence [13]. Additionally, restricting the specific genotypes detected by a screening test is being investigated as a strategy to increase clinical specificity of detecting disease with a likely decrease in clinical sensitivity [14].

Country-level data on HPV genotype carriage to inform screening algorithms, policy, and research are lacking [15]. There may be regional variable in the clinical utility for detection of specific HPV types. For example, HPV35 accounts for approximately 2% of invasive cancers globally [16] but up to 10% of invasive cancers in sub-Saharan Africa. Women of African ancestry in the United States are similarly more than twice as likely as women of other ethnicities to have HPV35 infections and more than three times as likely to develop HPV35-associated precancers [17]. Because HPV35 is not included in any currently available prophylactic HPV vaccines, separate detection of HPV35 may have clinical utility in select populations.

### Technical challenges and opportunities for HPV testing

Establishing a new cervical cancer screening program or sustaining an existing program requires navigating technical differences to choose an appropriate collection device, transport media, and test. Many of the collection devices and transport media options on the market are proprietary and specific to a single test or have been validated and approved for use in a subset of available tests (Table 3). Moreover, accessible and sustainable HPV testing sits within the broader continuum of care, the regulatory landscape, and procurement processes. We summarize several pressing technical challenges and opportunities associated with HPV testing globally below and in Table 4.

**Table 3:** Collection device, collection media, and test compatibility.

			Hologic PreservCyt	BD SurePath	Abbott Cervi-Collect kit	Aptima Specimen Transport Media	Roche Cell Collection Medium	cobas PCR Cell Collection Media	BD HPV diluent co-collection media	Digene specimen transport media	careHPV Collection Media	Trueprep AUTO Trnasport Medium	Dry swab	Solid transport card	Generic, U, or unspecified bufer
HPV and/or cytology compatible			Both	Both	HPV	HPV	Both	HPV	HPV	HPV	HPV	HPV	HPV	HPV	HPV
Compatible provider-collection brushes **			Broom-type or brush/ spatula ^a^	Broom-type or brush/ spatula ^a^	Cervi-Collect swab	Aptima Cervical Specimen Collection Device	Rovers Cervex ^a^	Rovers Cervex ^a^	Rovers Cervex ^a^	digene HC2 DNA Collection Device	*care- Brush*	FLOQ- swabs		Not spec-ified	
Compatible self-collection brushes			Qvintip, SelfCerv, Cobas Uni Swab	Cobas Uni Swab							care- Brush **		Qvintip, HerSwab, Viba Brush, FLOQ- swabs, Evalyn Brush**	Not spec-ified	HerSwab, Evalyn Brush, Delphi screener (unspec.); FLOQ-swabs (U)
*Test mfgr*.	*Platform*	*Assay*													
Abbott	m2000 sp/rt	*Real*Time High Risk (HR) HPV											[60–63] *VALHUDES*		[60]
Abbott	Alinity m	Alinity m High Risk (HR) HPV													
Hologic	Panther	Aptima HPV^b^		**		**									
Hologic	th												[61,64,65] *VALHUDES*	FTA Elute [66,67]	[60]
BD	Viper LT System	Onclarity HPV Assay		V4 [68]									[61,69] *VALHUDES*		
Cepheid	GeneXpert	Xpert HPV											[70,71] *VALHUDES*		
LifeRiver Biotech	Aurtrax extractor + Life 96 PCR System	Harmonia HPV, Venus HPV kit		V4e											unspecified
QuanDx	thermal cycler	MeltPro High Risk HPV Genotyping Assay											[72]		unspecified
															TE [72]
Atila Biosystems	thermal cycler	Ampfire HPV	**	**									[73]		unspecified
Genomica	NEDxA	CLART HPV 4	**	V4e[68] **						**			**		
Molbio	Truelab PCR analyzer	Truenat HPV-HR										**			
SeeGene	NIMBUS/STARlet, thermal cycler	Anyplex II HPV HR Detection	**	V4 [68] **	**								[65,71] *VALHUDES*		

Dark blue: Approved for use per UNITAD Cervical Cancer Technology Landscape [8] or

**included in indicated product insert, instructions for use, or manufacturer report (S1 Table); light blue: Evaluated in indicated studies (note: Included studies are not exhaustive).

^a^Alternative broom-type cervical swabs or combination swab/spatula collection devices can be used.

^b^ Aptima Hologic HPV is an mRNA test; the remainder of tests included detect DNA.

V4: Included in Valgent4 protocol with unprocessed cervical samples [68].

V4e: Extracted DNA from cervical samples included in Valgent4 protocol [68].

VALHUDES: Expected to be included in the VALHUDES protocol [74].

**Table 4:** Summary of technical challenges and opportunities for HPV testing globally.

Challenges	Opportunities
*Sample collection*	
• Collection devices and collection media are not standardized and validation of existing products across different testing platforms is limited • Proprietary devices optimized for sample collection are costly • Generic devices may increase complexity of lab processes • Liquid cytology media is too toxic to send into homes, incompatible with some platforms, has high cost and lab complexity • Liquid molecular buffers leave samples more susceptible to degradation in transit, have relatively high cost, and restrict sample usage to certain testing platforms • Dry swab collection leaves samples most susceptible to degradation in transit, risking false negatives	**•** Standardize a common buffer or sample type across existing HPV testing platforms **•** Expand validation studies of a wide range of buffers or sample types across platforms **•** Validate dry swab stability and clinical sensitivity for high-grade disease across HPV testing platforms
*Point-of-care tests*	
• HPV tests on the market remain too complex for true point-of-care use	**•** Expand near-patient or point-of-care testing approaches **•** Innovate true point-of-care testing platforms, especially leveraging recent SARS-CoV-2 rapid molecular tests
*Increasing access with self-collection*	
• Self-collected samples need to be transported from the screening participant to the lab • Results need to be communicated back to the screening participant with appropriate linkages to follow-up care • Technical challenges with processing vaginal instead of cervical samples • Cut points and limits of detection may not be optimal for dry swabs • Trained personnel are needed to support self-collection and follow-up	**•** Increase dry swab stability for transport **•** Adjust lab protocols for vaginal sample processing **•** Optimize test characteristics for compatibility with dry swabs **•** Characterize dry swab stability through multiple modes of transport **•** Integrate quality control indicators into samples and/or tests
*Clinical role of triage tests*	
• HPV DNA tests have low positive predictive values • Resource constraints dictate triage test used	**•** Investigate clinical role of extended HPV genotype-based risk stratification and other single-test strategies for screening and triage **•** Evaluate clinical performance of recent cervical imaging innovations
*Access to regulatory support and funding*	
• Many HPV tests on the market are not appropriately validated • Clinical evaluations of HPV tests employ variable study designs and sample types	**•** Create a standardized and well-validated set of reference samples
*Impacts of the COVID-19 pandemic*	
• People have inadequate access to affordable and suitable HPV testing platforms globally • Technology developed to mitigate the COVID-19 pandemic have exacerbated global health inequities	**•** Develop regional approval processes to facilitate access to new technologies **•** Leverage innovations for SARS-CoV-2 molecular detection to develop new HPV tests **•** Utilize centralized COVID-19 testing infrastructure for HPV testing **•** Implement decentralized sampling strategies, including at-home self-collection
*Procurement*	
• Per-test costs remain too high for widespread use without negotiated bulk pricing in LMICs • Many distinct and overlapping funding efforts are involved in test platform and consumables procurement	**•** Develop national essential diagnostics lists to integrate procurement at the governmental level **•** Organize a procurement and coordination effort, subsidized cartridges, and/or reagents for existing multi-analyte platforms

#### 2.1 Sample collection

*2*.*1*.*1 Self-collection for HPV testing*. One of the important advantages of HPV testing is that women can self-collect a cervicovaginal specimen [2], which has been currently incorporated into several international and country-level guidelines [18,19]. Self-collected sampling does not require a clinic visit and a pelvic exam with a speculum, which are necessary for cervical cytology or VIA. Self-collection can overcome barriers such as limited access to health facilities, shortage of personnel to perform pelvic evaluations, taking time off from work or home, transportation of the health facilities, embarrassment, stigma, pain, and discomfort associated with routine gynecological screenings, etc [20]. Moreover, self-collection has been widely demonstrated to improve screening uptake, especially when health systems adequately consider factors that improve efficacy of self-collection, such as developing culturally sensitive health education materials, financing and policies to ensure access and availability, male engagement, peer advocacy, engagement of community health workers, and promotion of self-efficacy and self-care [21,22].

Importantly, a 2018 meta-analysis of well-controlled, research studies found the clinical performance of HPV testing using self-collected cervicovaginal specimens was comparable to provider-collected cervical specimens when a PCR-based DNA test is used and slightly less sensitive when a signal-amplification DNA test is used [22]. RNA tests also appear less sensitive on self-collected specimens than provider-collected specimens, with no detected differences across collection devices or storage media [22].

However, clinical performance of HPV testing of self-collected specimens in real-world settings may have reduced performance [19] and may benefit from further optimization [23]. Current HPV testing protocols are based on sampling the cervix directly by the provider, who then typically places the cervical specimen into liquid-based cytology medium or “PCR” testing medium. By contrast, self-collection collects sloughed cervical and vaginal cells and mucus secretions from the fornix of the vagina and may also be “contaminated” with vaginal wall sampling. As a consequence, the composition of two specimens may differ, including the ratio of HPV-infected vs. HPV-uninfected epithelial cells and the amount of interfering mucus secretions. Thus, accuracy of HPV testing using self-collected specimens may be further improved by optimizing pre-analytical conditions such as the specimen collection (sampling device), storage (e.g., dry vs. liquid medium, what kind of medium, etc.), and handling (e.g., time from collection to testing and storage temperature) as well as the testing protocol (e.g., lysis and extraction methods, testing medium, and volume and cellularity of the specimen [19,23].

**Fig 2:**
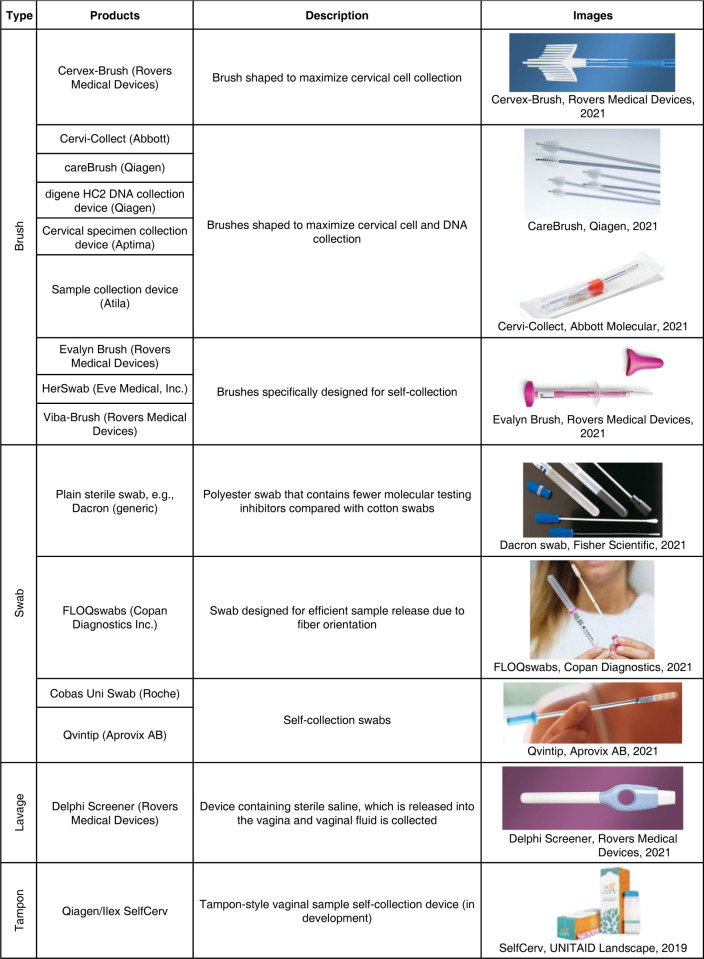
Selected cervical sample collection devices for HPV testing.

*2*.*1*.*2 Self-collection implementation considerations*. A major challenge associated with HPV test implementation is a lack of standardization of sample collection brushes and media [23]. The sheer number of options of collection devices (selected options shown in Fig 2) can be challenging for implementers [24]. A summary of self-collection studies using different collection media, sampling devices and tests, was published in 2018 (see article’s supplementary data) [22]. Yet, there are few or no comparative data on women’s acceptability and preference for self-sampling devices.

The choice of collection device has implications for downstream laboratory testing, as the amount of genetic material and inhibitors varies based on geometry and material of the collection device. Moreover, commonly available materials used for sample collection, like cotton swabs and tampons, may introduce additional inhibitors and require more complex laboratory procedures to recover HPV DNA for testing [25].

Aside from the sampling product, samples can be collected into liquid cytology buffers, liquid molecular buffers, or stored without a buffer (“dry”) (Table 5). Benefits of using liquid cytology media include specimen stability during transport and utility with both HPV and cytology testing. Drawbacks of liquid cytology media include high alcohol or formaldehyde content and large volumes of media [24] too toxic to send into homes for self-collection [26], incompatibility of some proprietary media and certain testing platforms, relatively high cost, challenging waste disposal, and additional laboratory processing complexity. Benefits of using liquid molecular buffers include cell lysis upon collection, allowing for simpler laboratory processing protocols, as well as moderate sample stability in transport. Drawbacks of liquid molecular buffers include relatively high cost and incompatibility of some proprietary media and certain testing platforms. Finally, benefits of dry swab collection include possibility of at-home collection without the presence of a toxic buffer, high flexibility to elute into a test-compatible buffer in the laboratory, and relatively low cost. Drawbacks of dry swab collection include a lack of preservative and relatively high susceptibility to time and temperature fluctuations in storage [24,27]. These drawbacks could reduce sample quality and pose the risk of false negatives and invalid results—especially with self-collection—without further optimization [19,23,27].

**Table 5:** Selected sample transport media for HPV testing.

Category	Compatible methods/analytes	Formulations*	Products
Liquid cytology	Cytology Conditionally: HPV DNA, HPV RNA, HPV oncoprotein	Preservative buffers containing methanol; sample conversion into molecular-friendly testing buffer frequently required	PreservCyt (Hologic)
			Cell collection medium (Roche)
		Preservative buffer containing formalin that crosslinks DNA-protein, which can be undone by boiling	SurePath (BD)
Liquid molecular	HPV DNA and/or RNA	Lysis buffer containing guanidine thiocyanate for DNA preservation	Cervi-Collect Kit (Abbott)
			Trueprep AUTO Transport Medium (Molbio)
		Lysis buffer containing lauryl sulfate lithium salt for RNA preservation	Specimen Transport Media (Hologic)
		Lysis buffer containing Tris/Sodium Chloride with surfactant for lysis and a DNA preservative	HPV diluent co-collection media (BD)
		Lysis buffer containing guanidine hydrochloride for DNA preservation	cobas PCR Cell Collection Media (Roche)
			digene Specimen Transport Medium (Qiagen)
			careHPV Collection Media
Dry	HPV DNA	Swab is collected and placed into a sterile tube in the absence of media	Dry swab
		Cards treated with lysis solution and color-changing dye to indicate sample is applied (e.g., PK 226 contains ionic detergent for lysis)	Solid transport cards, e.g. PK 226 Paper (PerkinElmer)
Other buffers	Dependent on buffer	Buffered media generally used for viral preservation, compatible with nucleic acid amplification testing	Universal Transport Media (U, Copan)
		Some tests indicate compatibility with suspended cervical cells without specifying suspension buffer[8]	Generic/unspecified

* Adapted from manufacturer reports (S1 Table).

Currently, there is not a single universal molecular testing buffer. An ideal universal molecular buffer for testing for HPV and other analytes should be intended for molecular testing for any HPV target, including DNA, mRNA, and perhaps even proteins. It should be inexpensive and non-toxic, stabilize the molecular targets under a wide range of environmental conditions, and require minimal laboratory processing. Several technical challenges persist even when a buffer is designed for molecular testing. Many molecular testing buffers include lysis agents, including salts or detergents, that preserve DNA in a range of storage conditions, but that also interfere with enzymatic activity in downstream DNA detection. Different testing platforms may use different methods to purify DNA from the buffers’ inhibitory components, thereby limiting the universality of molecular lysis buffers in use today. There is a technical knowledge gap on protecting DNA from degradation through time and temperature fluctuations without requiring DNA extraction and/or purification during sample processing.

There is also limited validation among the many products that already exist for sample collection, which provides technical barriers for implementers. Several combinations of collection buffers and devices have been evaluated across HPV testing platforms [22], but there is not yet sufficient evidence to conclude that a specific buffer or collection method is ideal for widespread use, nor is there sufficient validation of common buffers across platforms. Building from the UNITAID Cervical Cancer Technologies Landscape [8], compatibility of collection devices, transport media, and HPV tests are presented in Table 3.

Reducing the complexity of the testing landscape could include (1) standardizing a common buffer or sample type across platforms and (2) expanding validation studies of a wide range of buffers or sample types across platforms. Validation studies investigating dry swab stability and resulting clinical sensitivity for high-grade disease across platforms could be useful in expanding the use of dry swabs.

*2*.*1*.*3 Increasing access with self-collection*. Self-collection is a safe, easy, and acceptable method of sample collection that allows for decentralized cervical cancer screening participation for women who otherwise may not participate. However, there are several challenges specific to self-collection. Logistically, samples need to be transported from the screening site to the laboratory, and the result of the test needs to be communicated back to the screening participant with appropriate linkages to follow-up care [28]. One example of an effective program with text message-based notifications is Program ROSE in Malaysia [29]. Moreover, there are technical challenges associated with processing self-collected vaginal samples compared with provider-collected cervical samples, including physical sample composition and non-standardized, non-automated sample preparation protocols [28]. If self-collected samples are collected and transported dry, then sample preparation protocols and tests cut-points and limits of detection may need to be optimized to maintain high clinical sensitivity and specificity found with tests currently approved for provider-collected samples [23]. Finally, trained personnel are needed to support community-based self-collection efforts—including administering self-collection kits and educating participants about self-collection—and to provide follow-up care [28].

Additional efforts to optimize dry swab stability for transport, protocols for vaginal sample processing, and test characteristics for compatibility with dry swabs could improve performance of self-collection with dry swabs. Characterization of sample stability in diverse modes of transport (e.g., through the mail system or transport by courier) over different time spans is needed to inform future self-collection schemes. Moreover, quality control indicators should be designed at the sample and/or test level to identify sample degradation in dry swabs.

*2*.*1*.*4 Emerging evidence on urine*. In addition to swab-based testing, there is growing body of evidence of good analytic performance of HPV testing using urine. A recent meta-analysis found that, like using self-collected cervicovaginal specimens, using a PCR-based HPV test achieved better performance than other technologies for HPV detection and approached that of a provider-collected cervical specimen [30]. The meta-analysis also found accuracy (vs. the provider-collected cervical specimen) improved using first void vs. other collections and with preservation of the specimen using the Colli-Pee (Novosanis, Wijnegem, Belgium) or other preservatives vs. without. However, the number and size of studies have been small and with significant heterogeneity in settings, design, and rigor [30]. There is also a lack of well-powered studies in the intended use population with endpoint of cervical precancer and cancer to confirm clinical performance. Finally, there are critical methodologic and logistical issues for HPV testing of urine that need to be addressed in order to translate in its use in controlled research settings to scaled-up use in real-world settings. Like for self-collected cervicovaginal specimens, these include its stability in ambient conditions given the unknown interval between collection and testing and the handling and transportation to the specimen to the testing.

#### 2.2 Point-of-care tests

Currently, HPV tests on the market remain too complex or too expensive for true point-of-care use, which would enable rapid screening testing, diagnose, and treat patients within a single appoinent, thereby reducing loss to follow-up [31]. Expanding near-patient or point-of-care testing approaches, like Xpert HPV (Cepheid), would enable more widespread testing than with centralized laboratory platforms alone. Additional innovation is needed for true point-of-care testing platforms with reduced cost, infrastructure requirements, and time-to-result [32]. Recent work on molecular point-of-care tests developed for SARS-CoV-2 can be leveraged to develop point-of-care HPV tests, as discussed in section 2.6. Point-of-care tests using self-collected samples have the potential to increase coverage of cervical cancer screening, especially among specific populations who currently do not have access to screening services. Significant invesent would be needed to implement testing units in multiple primary health centers, and programs would require a substantial effort to train personnel to carry out the remainder of the care cascade.

#### 2.3 Waste management

Perhaps an underappreciated challenge to expanding the use HPV testing and other *in vitro* diagnostics globally, especially in SSA [33], is the management and disposal of the medical waste generated from testing. Waste toxic buffers and the vials that contain them, potentially contaminated/biohazardous specimens, disposable plastic testing materials—such test cartridges, pipette tips, etc.—represent unique challenges to many LMICs that do not have the necessary regulations, resources, infrastructure, or effective means to manage and dispose of them. Novel HPV testing systems are needed that are not only engineered to be user-friendly but environmentally-friendly, as well, in order to achieve a sustainable screening program and mitigate any unintended harms to human life as the result of waste mismanagement [33,34].

#### 2.4 Access to regulatory support and funding

Despite the high number of HPV tests on the market, very few of them have been properly validated. Intensive resources and effort are required for implementing new validation studies. Existing sample banks limit test developers to samples that are stored in specific buffers or are stored dry. Both new studies and existing sample banks may not include appropriate correlated data on HPV status and clinical endpoints. Test developers could benefit from a standardized and well-validated set of reference samples. Even though the creation of validated banks of samples also demands significant invesent of time, resources, and effort, having such bank could facilitate the validation of novel HPV tests from small companies with innovative and promising technologies.

#### 2.5 Procurement and sustainability

Even with new innovations and further validation of existing tests, access to HPV testing in LMICs will require a global procurement strategy [35,36]. Such global procurement strategy has been successful for vaccines though the GAVI alliance. Per-test costs will likely remain too high for widespread use without negotiated bulk pricing at multi-national scales and subsidization, as is done for HPV vaccines by GAVI, as well as coordination among the many distinct and overlapping funding efforts in the global diagnostics market today [37].

For example, as of July 2021, the Clinton Health Access Initiative (CHAI) reports that per-test pricing for nine selected HPV tests ranged from US$4.95 through US$14.90 [38] (note: prices were reported by manufacturers and include different items—e.g. reagents only; reagents with controls, instrument, service, and maintenance; cost of invalid results; etc.—and may have a higher cost in practice). One-time instrument cost ranged from US$0 (i.e., included in per-test cost of tests) up to US$600,000 [38]. Currently, the Pan American Health Organization (PAHO) offers reduced pricing but needs to be evaluated per technology, including relative to the prices offered by local distributors, and is limited to WHO-prequalified tests [39]. Data on cost reduction and procurement system strengthening for Latin American through the PAHO Strategic Fund are forthcoming.

Experts have called for[40] and produced an essential diagnostics list (EDL) [41], which includes HPV DNA testing. Development of national EDLs could be a mechanism to catalyze integrated procurement at the governmental level. With an organized and coordinated procurement effort, subsidized HPV tests for existing multi-analyte platforms in LMICs could readily expand access to high-performance cervical cancer screening and prevention, and control of other diseases requiring IVDs. Importantly, availability of funding for sustainable HPV test procurement will be a crucial challenge to solve.

Finally, once procured, sustained use of HPV tests relies on the ability to repair equipment, continue to source reagents and test supplies, and troubleshoot questionable test performance [32]. Ongoing efforts to improve supply chains and local biomedical engineering infrastructure will play a major role in sustained access to HPV testing.

### 3. Triage following HPV screen-positive results

Managing screen-positive results requires a second test or evaluation in which clinicians determine whether a patient should be treated, surveilled for a period of time, or returned to regular screening intervals. While screening tests should be highly sensitive at the cost of specificity, triage tests should prioritize specificity to determine the appropriate clinical course for the patient. With many existing technologies, managing screen-positive results requires a follow-up visit and a second test or a visual or imaging-based assessment, and current methods including colposcopy and pathology are challenging and resource-intensive to implement in resource-limited settings. Triage tests should be user-friendly and affordable, and as point-of-care HPV screening tests are developed and become more widely available, a triage method that can be completed immediately following an HPV-screen positive result, within the timeframe of a single appointment, should be prioritized to reduce loss-to-follow-up.

HPV DNA tests have excellent negative predictive value (est. >99%) and low positive predictive value (est. <20%) [42]. In high-income settings, HPV DNA screen-positive tests are usually followed by cytology and/or partial genotyping of HPV16/18 as a triage test and histologic diagnosis prior to treatment. In resource-constrained settings, HPV DNA screen-positive tests are generally followed by partial genotyping of HPV16/18, VIA, or visual assessment for treatment (VAT), depending on resources available [43].

Potential strategies to triage HPV DNA screen-positive women are being investigated, including detecting HPV biomarkers more specific to disease progression such as HPV mRNA [44,45], HPV E6/E7 oncoproteins, markers of HPV-induced cell cycle alterations (e.g., Ki-67 and *p*16^ink4a^) [45–47], host or viral DNA methylation; extended HPV genotyping [45,46]; and HPV viral load [44]. It may not be logistically or financially feasible to run a second test for triage, especially in low resource settings; therefore, strategies like limited or extended HPV genotype-based risk stratification may be most appropriate to minimize total clinic visits.

The WHO already recommends partial genotyping of HPV16 and HPV18, and in some cases HPV45, for the management—not screening, per se—of HPV-positive women. HPV16- or HPV18-positive women are at sufficiently high risk that they recommended further evaluation and treatment if needed regardless of the cytology result [2]. Increasingly, the role of extended or full HPV genotyping for management of HPV-positive women is being investigated [13,48].

Recent cervical imaging innovations may provide technological solutions for enabling high-performance diagnosis for screen-positive women. Automated visual examination (AVE), a machine-learning algorithm that analyzes images to improve accuracy of visual cervical inspection, may increase access to high-performance diagnosis; even though AVE showed very promising initial results, it still requires further development and validation before its implementation in clinical practice [49].

Additionally, the high-resolution microendoscope (HRME) is an emerging technology that provides subcellular resolution for real-time cervical imaging [8,32]. The HRME hardware can be paired with a laptop, single-board computer, tablet, or mobile phone, and similarly to AVE, HRME software automates image processing to aid clinical decision support, improving diagnostic accuracy [32].

Once rigorously evaluated, automated imaging modalities with HPV genotyping may provide the highest quality determination of who should be preventively treated [49]. Procurement and implementation of imaging technologies will likely be more feasible when integrated into existing, available devices, such as cell phones.

### 4. Impacts of the COVID-19 pandemic

Access to more affordable and suitable testing platforms is needed. The massive disruptions to global and national health systems brought by the COVID-19 pandemic showed the need for new tests suitable for all resource-settings. Importantly, diagnostic technology development and access have exacerbated global health inequities, with manufacturing and testing capacity lacking where it has been needed most [50].

In response to SARS-CoV-2 testing demand that outpaced supply, several initiatives were launched [51]. Similarly, the U.S. NIH launched the Rapid Acceleration of Diagnostics (RADx, https://www.nih.gov/research-training/medical-research-initiatives/radx/radx-programs) initiative to speed test development, commercialization, and implementation of SARS-CoV-2 testing. RADx focuses on multiple areas, including point-of-care and home-based tests, and testing strategies to reduce health disparities, and increased testing capacity with high-throughput platforms. Emergency measures were taken in most countries to expedite the approval process to get vaccines or drugs into their countries. Building from these measures, regional approval approaches could facilitate access to new technologies.

As a result of the pandemic, an unprecedented number of molecular tests came onto the commercial market, ranging from home-use molecular tests through ultra-high-throughput platforms. As of November 2022, FIND (https://www.finddx.org/test-directory/) has tracked 2,099 commercialized molecular tests, 196 of which are categorized as near point-of-care, and 1,043 are categorized as true point-of-care. The tests included in the directory have different regulatory statuses, which are detailed in FIND’s directory, with 1,465 tests approved under European CE marking for in vitro diagnostics (CE-IVD) and WHO Emergency Use Listing (EUL) guidelines. These new technology platforms that enable molecular testing at home, at the point-of-care in decentralized clinics, or in centralized labs with samples collected at home all create major opportunities for expanded HPV testing. One clear opportunity is to develop HPV assays into the platforms that were developed, manufactured, and commercialized for SARS-CoV-2 detection. Another is to apply the same design principles to develop new molecular tests specific to HPV DNA, a simpler target to detect compared to SARS-CoV-2 RNA, and therefore potentially at a lower cost.

In addition to home-based and point-of-care testing, a major opportunity that arose from the COVID-19 pandemic was the expansion of centralized molecular testing. Major diagnostic labs globally acquired new molecular testing capabilities for COVID-19 [52,53]. The infrastructure, testing platforms, and technical knowledge associated with the expansion of molecular testing capability can be leveraged to implement HPV DNA testing within the same laboratories if, and when, HPV DNA assays are developed for the existing platforms. Along the same lines, the pandemic has allowed us to rethink sampling strategies, with at-home self-collection mailed to a centralized facility as an option to increase access to testing.

Moreover, there is now more molecular testing infrastructure and support globally; for example, the Africa Centers for Disease Control coordinated and promoted political will for molecular testing, facilitated collaborations and integration across the continent, and facilitated open data sharing. Additionally, the African Society of Laboratory Medicine facilitated sharing best practices and online trainings for molecular testing through digital platforms The support infrastructure built in response to the COVID-19 pandemic can be expanded to support other critical health programs, like HPV DNA testing.

## Other considerations

Secondary cervical cancer prevention by HPV testing is a critical component of the WHO strategy to eliminate cervical cancer as a public health problem. Several technical challenges currently limit access to cervical cancer screening. Expanding validation of self-collection devices, sample storage media or dry storage, and HPV testing platforms could be readily solved with invesent in new validation studies. Additionally, targeted efforts to leverage recent technological advances, close to home- and point-of-care SARS-CoV-2 tests, could enable faster development and scale-up of true point-of-care HPV tests. Solving these technical challenges will contribute to improved accessibility of HPV testing.

Notably, many challenges in cervical cancer prevention exist outside of HPV testing availability. For one, effective training for laboratory personnel, and quality control following implementation, are critical to accuracy and cross-contamination prevention. Moreover, once screening technologies are implemented, cervical cancer prevention programs may experience challenges in other steps of the care cascade. There will be a substantial increase in screen-detected high-grade precancerous lesions that will require diagnosis, treaent, and follow-up by trained providers with proper technology. Currently, there are weak healthcare infrastructure and a lack of healthcare providers trained in cervical cancer prevention in most LMICs with a high burden of cervical cancer. Context-appropriate and effective training programs, like the Gynecologic Oncology Global Curriculum and Mentorship Program of the International Gynecologic Cancer Society, provide a good model for training healthcare providers to respond to an increase in screen-detected cancers [54,55].

Additionally, certain populations of people, including transgender men, non-binary and intersex individuals who have a cervix, immunocompromised women, and women with drug-induced immunosuppression, are systematically underscreened for cervical cancer [2]. Addressing these disparities with additional research and resources will be an important priority for cervical cancer prevention.

To achieve the 90-70-90 goals, addressing challenges related to HPV technical barriers—as well as screening uptake, availability of triage tests, healthcare system capacity, health disparities, and appropriate treaent—will need creative, sustainable, and scalable solutions.

## Conclusions

Several technical barriers and opportunities will need to be considered to increase cervical cancer screening coverage to 70% globally. HPV testing allows for self-collection and samples and the possibility to decentralize screening. We recommend prioritizing: validating more combinations of sample collection buffers and tests for greater flexibility, developing a universal molecular buffer, investigating the role of HPV biomarkers for screening and management of screen-positives, and thoughtfully considering the context around HPV testing, including procurement and waste management.

## Supporting information

S1 Table Manufacturer reports. (DOCX)
