# Supplementary material for: Toward 70% cervical cancer screening coverage: Technical challenges and opportunities to increase access to human papillomavirus (HPV) testing
Source: PLOS Glob Public Health. 2023 Aug 16;3(8):e0001982. doi: 10.1371/journal.pgph.0001982 (PMC10431663; doi:10.1371/journal.pgph.0001982)
Supplement: S1 Table — (DOCX) [file pgph.0001982.s001.docx]

**Supplementary information**

**S1 Table:** Manufacturer reports

| **Company** | **Reference** | **Year** |
| --- | --- | --- |
| Abbott | Abbott Cervi-Collect Specimen Collection Kit Instructions for Use. | 2017 |
| Atila Biosystems | AmpFire HPV Genotyping Assay User Manual, Version 1.03. | -- |
| BD | Cervical Brush Collection Kit for the BD Onclarity HPV Assay, Rev. 07. | 2020 |
| Copan | U - Universal Transport Medium for viruses. | 2021 |
| Genomica | CLART HPV4: Genotyping of Human Papillomavirus via Genomic Identification for In Vitro Diagnosis, Version 12. | 2021 |
| Hologic | Aptima HPV Assay Package Insert, AW-12820 Rev. 002. | 2017 |
| Hologic | Safety Data Sheet: Transport Media/Collection Devices. | 2015 |
| Hologic | ThinPrep Pap Test PreservCyt Solution Instructions for Use, Rev. 001. | 2019 |
| Molbio | Safety Data Sheet: Trueprep AUTO Transport Medium for Swab Specimen Pack, Issue No. 04. | 2019 |
| Molbio | Truenat HPV-HR Pack Insert, Version 03. | 2021 |
| Qiagen | Safety Data Sheet: Specimen Transport Medium (S) CE, Version 1.0. | 2021 |
| Roche | Roche Cell Collection Medium, Doc Rev. 2.0. | -- |
| Roche | Safety Data Sheet: Kit cobas PCR Media Dual Swab 100 Pkt IVD, Version 1.4. | 2018 |
| SeeGene | Anyplex II HPV HR Detection. | -- |
